# Supplementary figures and images for: Regulation of inflammatory genes in decidual cells: Involvement of the bromodomain and extra-terminal family proteins
Source: PLoS One. 2023 Mar 10;18(3):e0280645. doi: 10.1371/journal.pone.0280645 (PMC10004631; doi:10.1371/journal.pone.0280645)

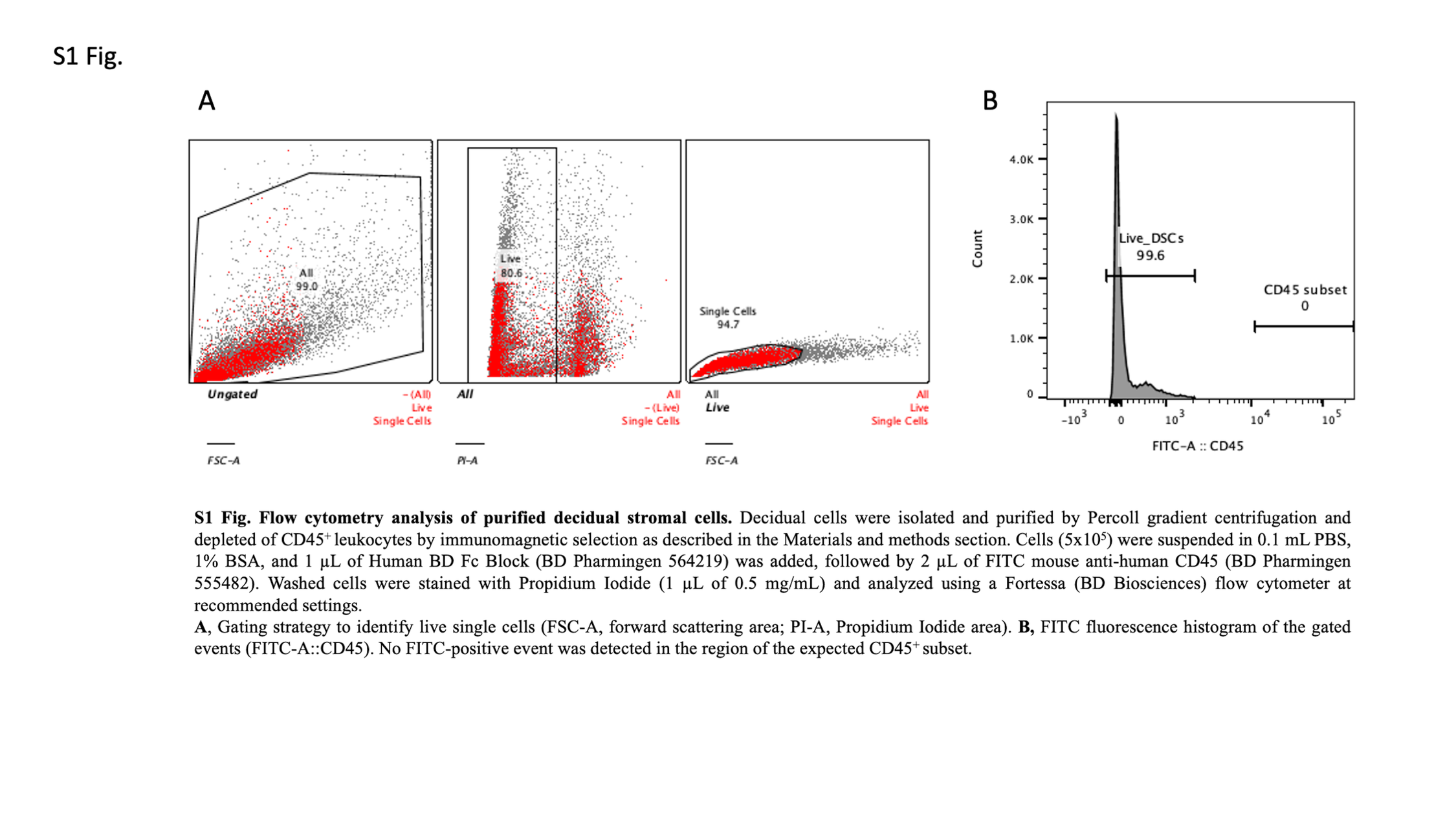

Supplement: S1 Fig — Decidual cells were isolated and purified by Percoll gradient centrifugation and depleted of CD45+ leukocytes by immunomagnetic selection as described in the Materials and methods section. Cells (5x10^5^) were suspended in 0.1 mL PBS, 1% BSA, and 1 μL of Human BD Fc Block (BD Pharmingen 564219) was added, followed by 2 μL of FITC mouse anti-human CD45 (BD Pharmingen 555482). Washed cells were stained with Propidium Iodide (1 μL of 0.5 mg/mL) and analyzed using a Fortessa (BD Biosciences) flow cytometer at recommended settings. A, Gating strategy to identify live single cells (FCC-A, forward scattering area; PI, Propidium Iodide). B, FITC fluorescence histogram of the gated events (FITC-A::CD45). No FITC-positive event was detected in the region of the expected CD45+ subset. (TIF) [file pone.0280645.s001.tif]

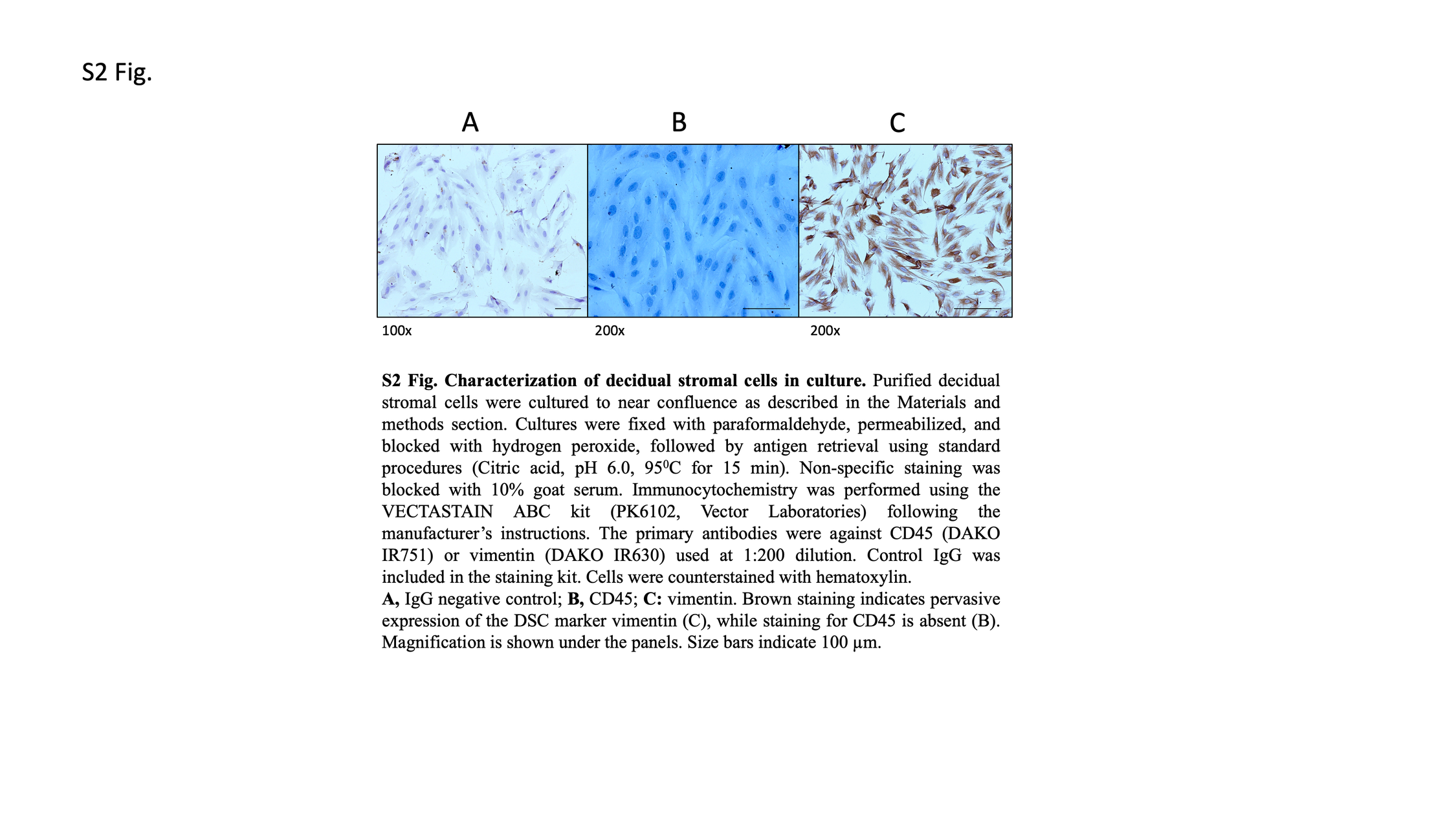

Supplement: S2 Fig — Purified decidual stromal cells were cultured to near confluence as described in the Materials and methods section. Cultures were fixed with paraformaldehyde, permeabilized, and blocked with hydrogen peroxide, followed by antigen retrieval using standard procedures (Citric acid, pH 6.0, 95C for 15 min). Non-specific staining was blocked with 10% goat serum. Immunocytochemistry was performed using the VECTASTAIN ABC kit (PK6102, Vector Laboratories) following the manufacturer’s instructions. The primary antibodies were against CD45 (DAKO IR751) or vimentin (DAKO IR630) used at 1:200 dilution. Control IgG was included in the staining kit. Cells were counterstained with hematoxylin. A, IgG negative control; B, CD45; C: vimentin. Brown staining indicates pervasive expression of the DSC marker vimentin (C), while staining for CD45 is absent (B). Magnification is shown under the panels. Size bars indicate 100 μm. (TIF) [file pone.0280645.s002.tif]

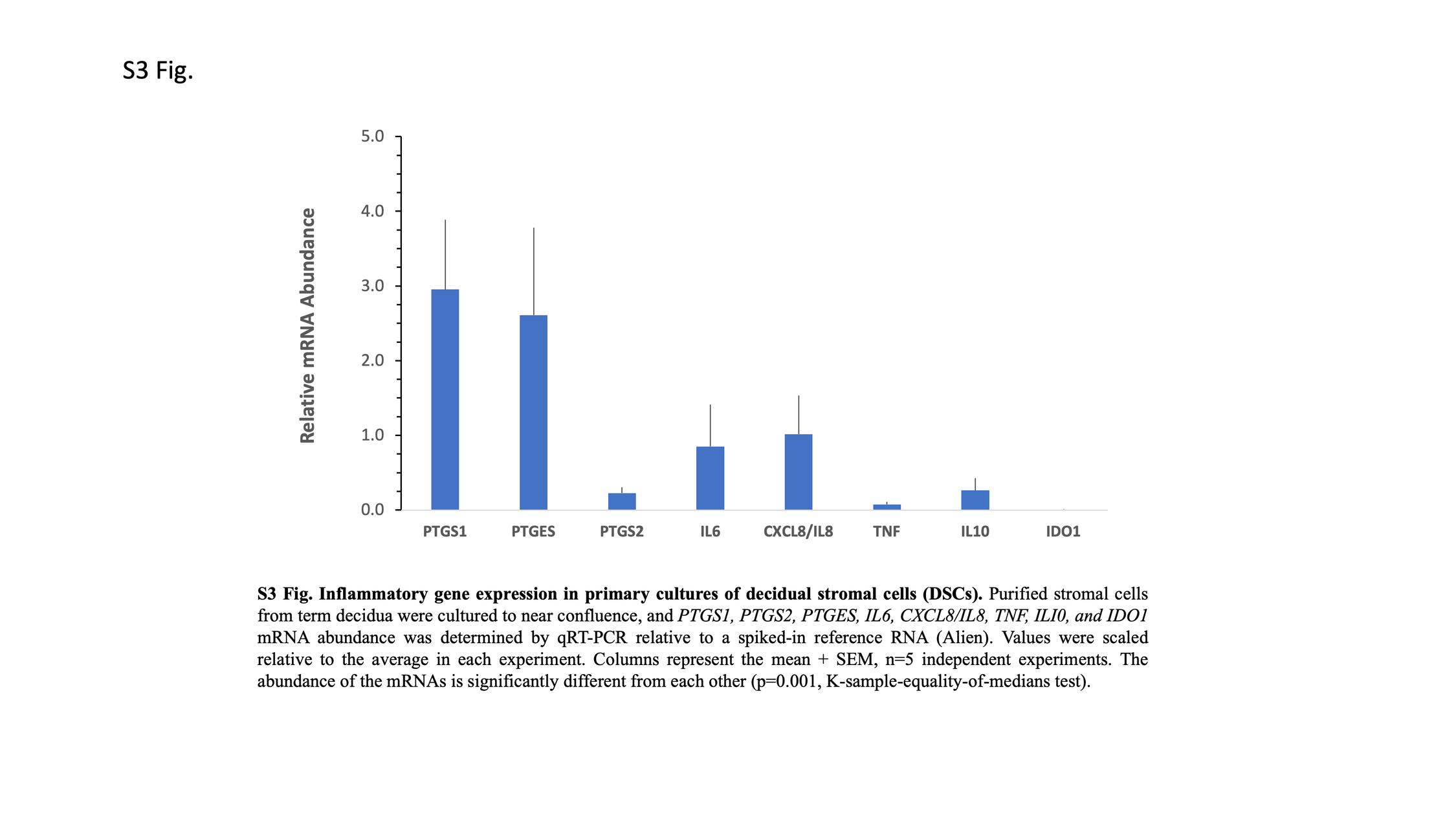

Supplement: S3 Fig — Purified stromal cells from term decidua were cultured to near confluence, and PTGS1, PTGS2, PTGES, IL6, CXCL8/IL8, TNF, ILI0, and IDO1 mRNA abundance was determined by qRT-PCR relative to a spiked-in reference RNA (Alien). Values were scaled relative to the average in each experiment. Columns represent the mean + SEM, n = 5 independent experiments. The abundance of the mRNAs is significantly different from each other (p = 0.001, K-sample-equality-of-medians test). (TIF) [file pone.0280645.s003.tif]

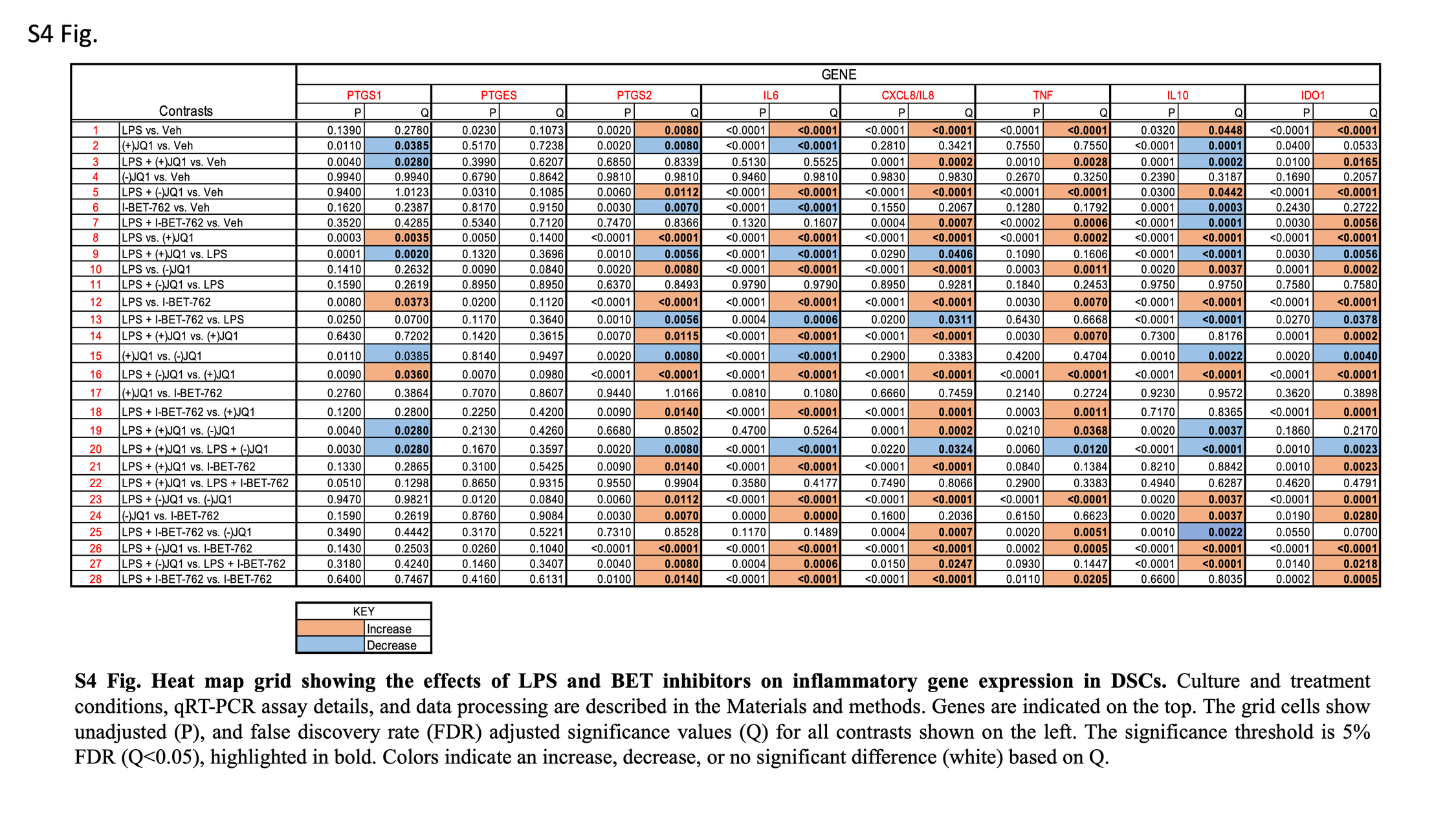

Supplement: S4 Fig — Culture and treatment conditions, qRT-PCR assay details, and data processing are described in the Materials and methods. Genes are indicated on the top. The grid cells show unadjusted (P), and false discovery rate (FDR) adjusted significance values (Q) for all contrasts shown on the left. The significance threshold is 5% FDR (Q<0.05), highlighted in bold. Colors indicate an increase, decrease, or no significant difference (white) based on Q. (TIF) [file pone.0280645.s004.tif]

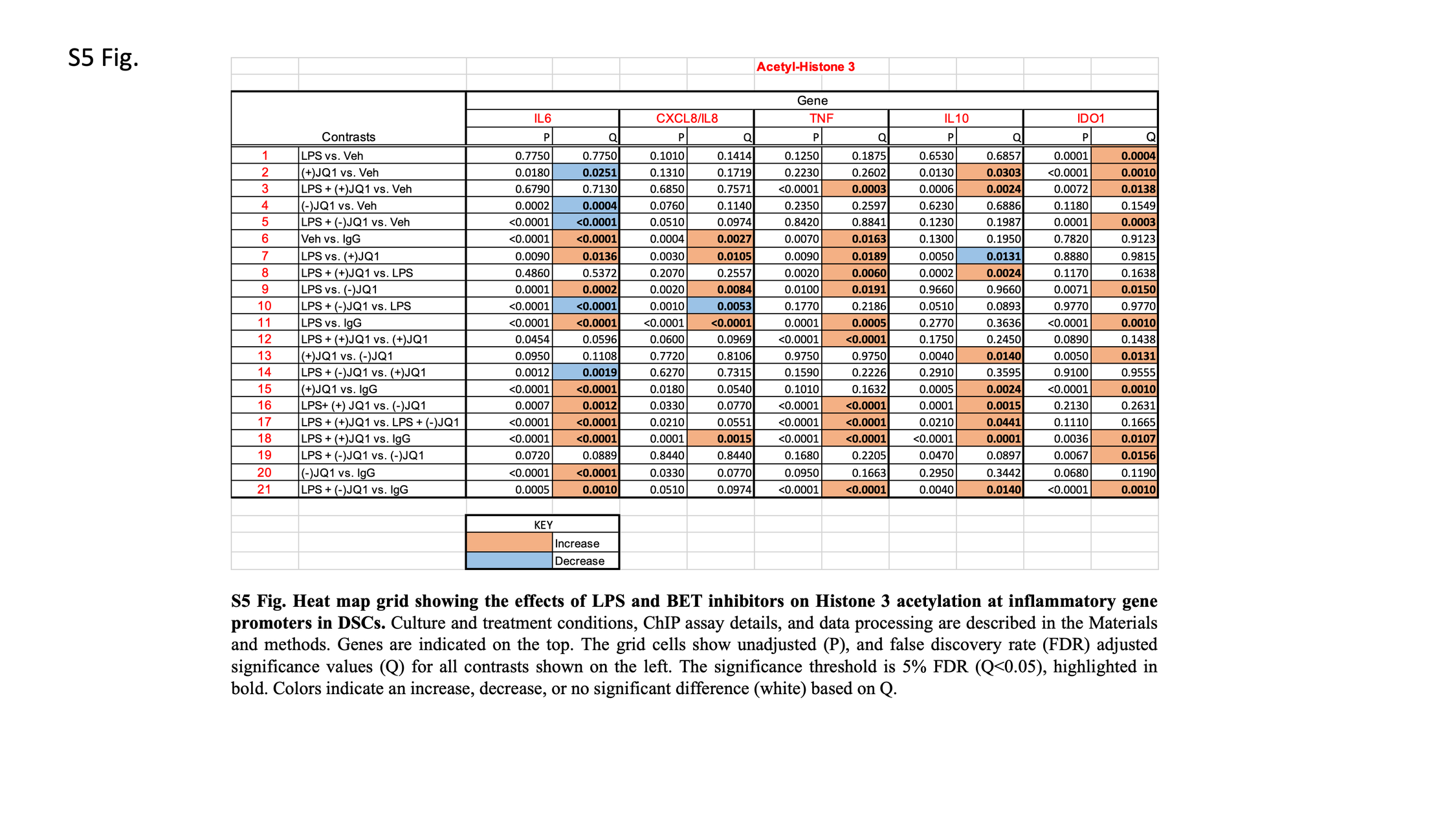

Supplement: S5 Fig — Culture and treatment conditions, ChIP assay details, and data processing are described in the Materials and methods. Genes are indicated on the top. The grid cells show unadjusted (P), and false discovery rate (FDR) adjusted significance values (Q) for all contrasts shown on the left. The significance threshold is 5% FDR (Q<0.05), highlighted in bold. Colors indicate an increase, decrease, or no significant difference (white) based on Q. (TIF) [file pone.0280645.s005.tif]

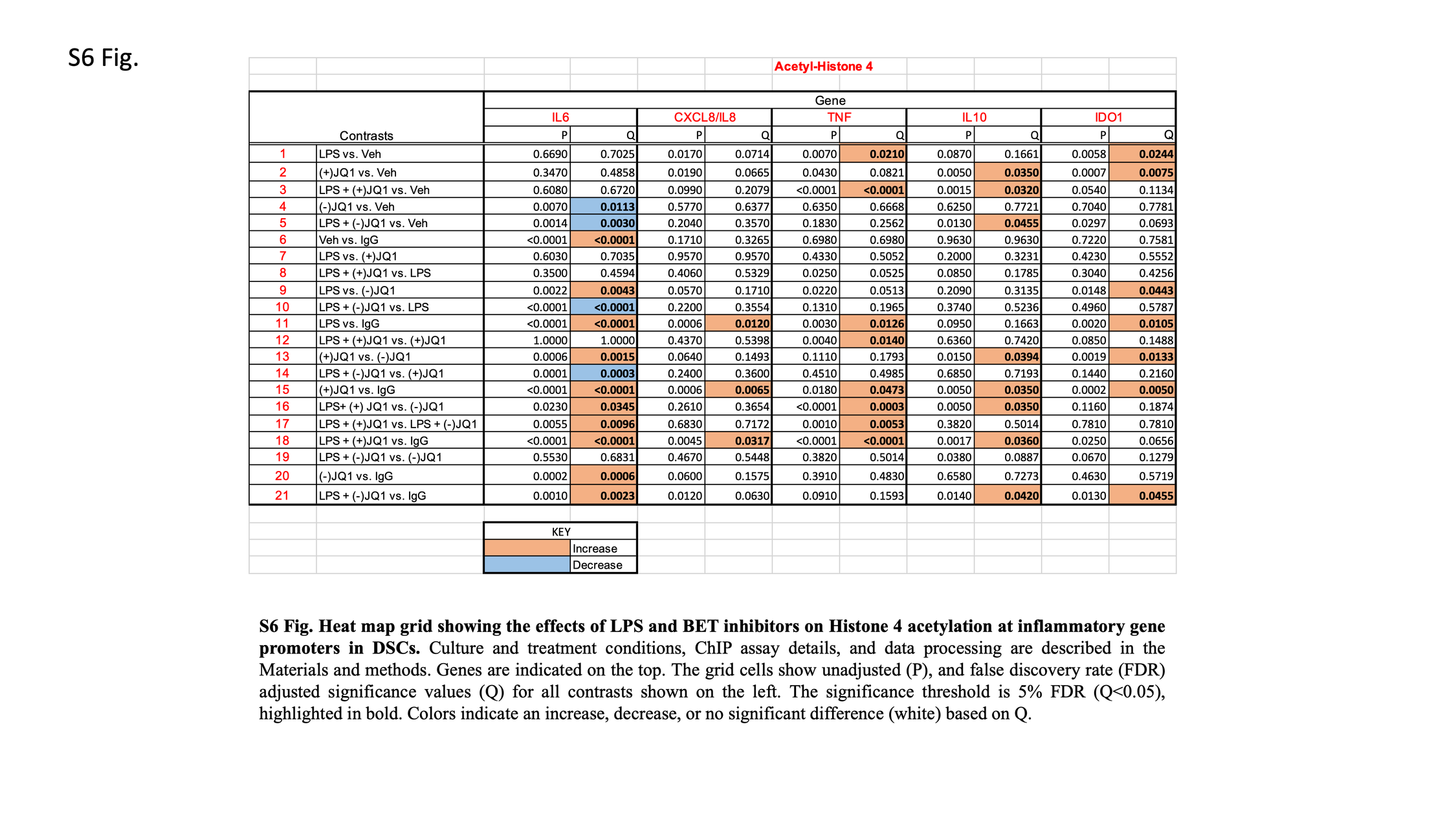

Supplement: S6 Fig — Culture and treatment conditions, ChIP assay details, and data processing are described in the Materials and methods. Genes are indicated on the top. The grid cells show unadjusted (P), and false discovery rate (FDR) adjusted significance values (Q) for all contrasts shown on the left. The significance threshold is 5% FDR (Q<0.05), highlighted in bold. Colors indicate an increase, decrease, or no significant difference (white) based on Q. (TIF) [file pone.0280645.s006.tif]

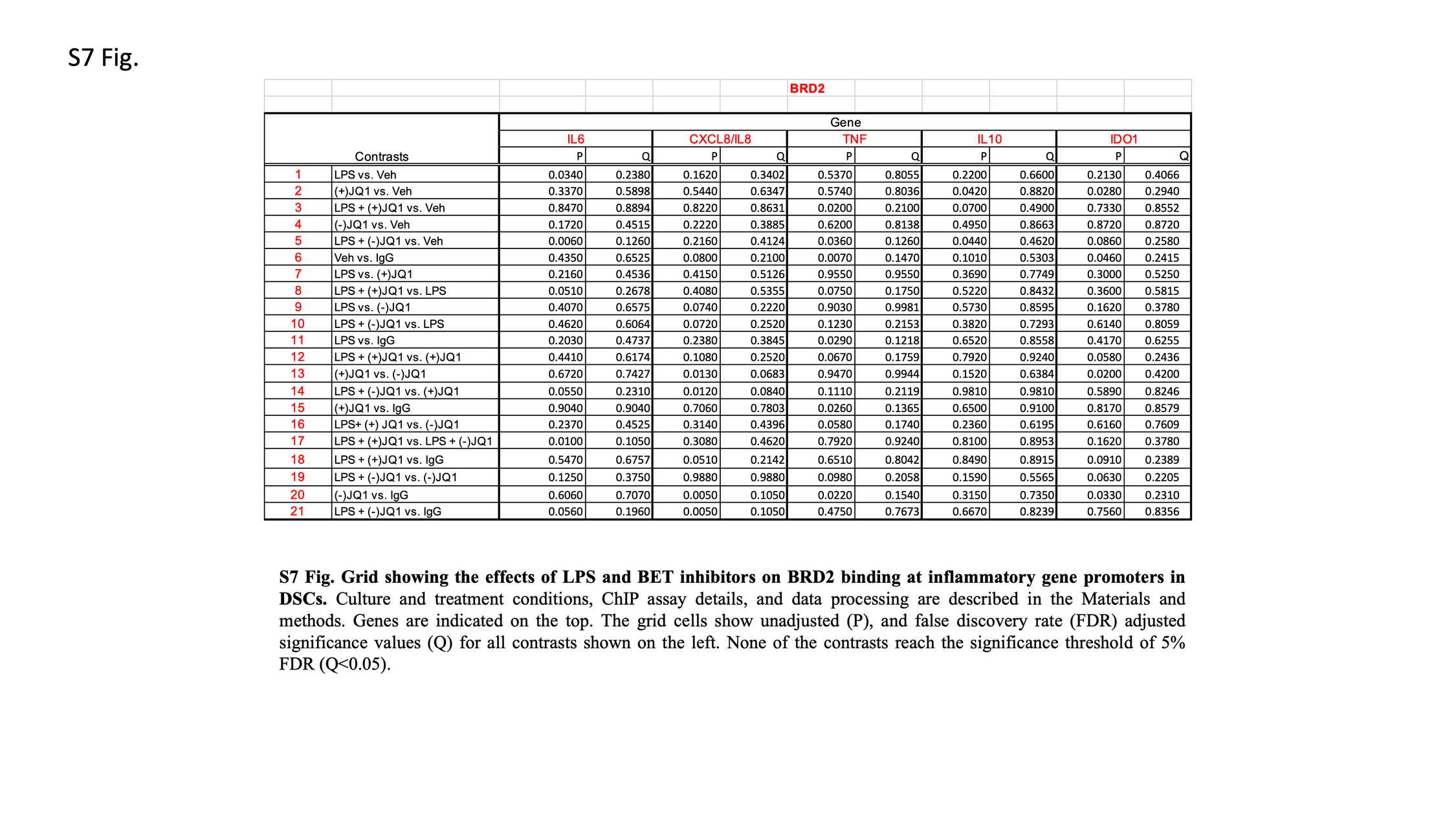

Supplement: S7 Fig — Culture and treatment conditions, ChIP assay details, and data processing are described in the Materials and methods. Genes are indicated on the top. The grid cells show unadjusted (P), and false discovery rate (FDR) adjusted significance values (Q) for all contrasts shown on the left. None of the contrasts reach the significance threshold of 5% FDR (Q<0.05). (TIF) [file pone.0280645.s007.tif]

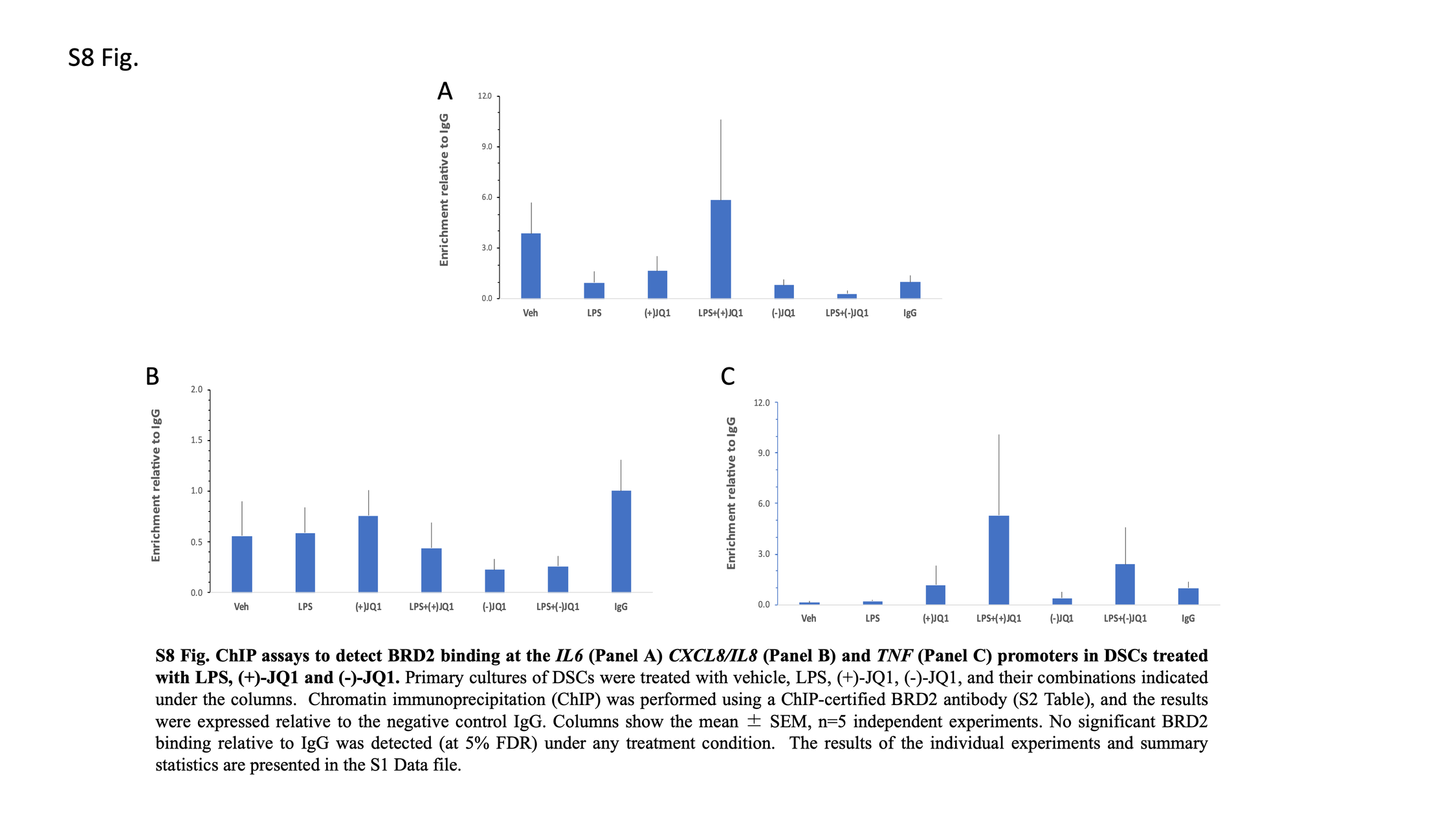

Supplement: S8 Fig — ChIP assays to detect BRD2 binding at the IL6 (Panel A) CXCL8/IL8 (Panel B) and TNF (Panel C) promoters in DSCs treated with LPS, (+)-JQ1 and (-)-JQ1. Primary cultures of DSCs were treated with vehicle, LPS, (+)-JQ1, (-)-JQ1, and their combinations indicated under the columns. Chromatin immunoprecipitation (ChIP) was performed using a ChIP-certified BRD2 antibody (S2 Table), and the results were expressed relative to the negative control IgG. Columns show the mean ± SEM, n = 5 independent experiments. No significant BRD2 binding relative to IgG was detected (at 5% FDR) under any treatment condition. The results of the individual experiments and summary statistics are presented in the S1 Data. (TIF) [file pone.0280645.s008.tif]

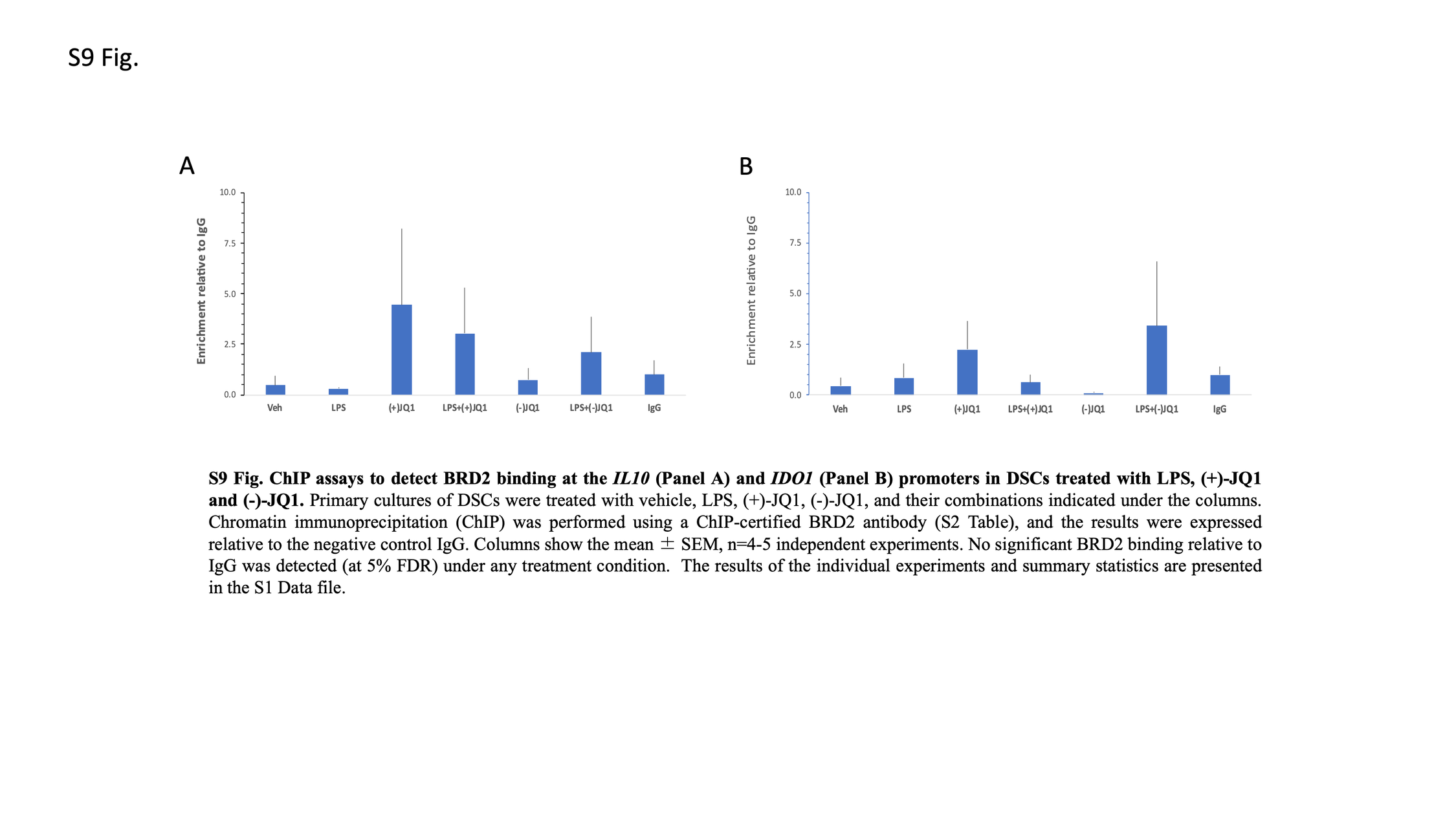

Supplement: S9 Fig — ChIP assays to detect BRD2 binding at the IL10 (Panel A) and IDO1 (Panel B) promoters in DSCs treated with LPS, (+)-JQ1 and (-)-JQ1. Primary cultures of DSCs were treated with vehicle, LPS, (+)-JQ1, (-)-JQ1, and their combinations indicated under the columns. Chromatin immunoprecipitation (ChIP) was performed using a ChIP-certified BRD2 antibody (S2 Table), and the results were expressed relative to the negative control IgG. Columns show the mean ± SEM, n = 4–5 independent experiments. No significant BRD2 binding relative to IgG was detected (at 5% FDR) under any treatment condition. The results of the individual experiments and summary statistics are presented in the S1 Data. (TIF) [file pone.0280645.s009.tif]

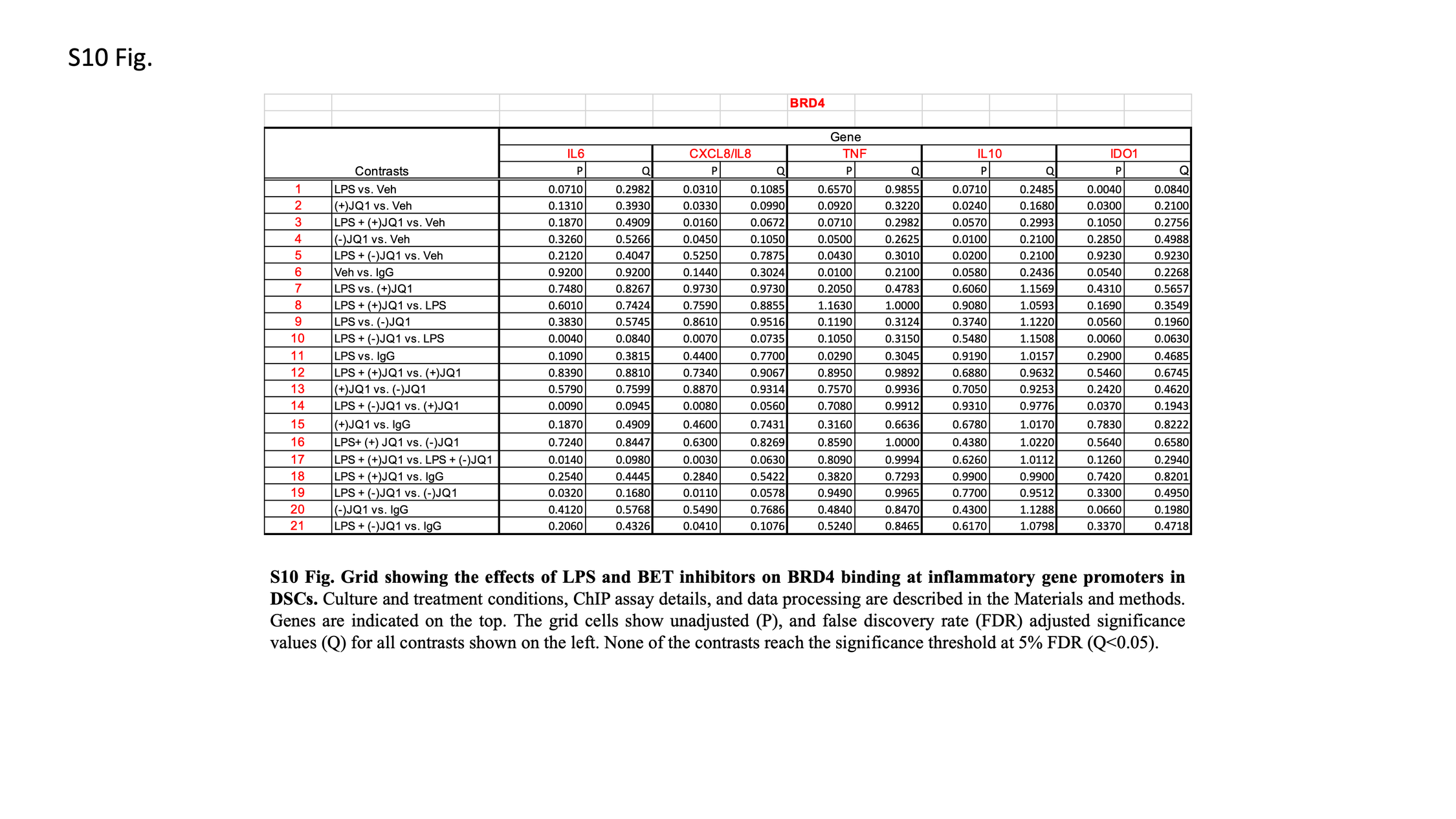

Supplement: S10 Fig — Culture and treatment conditions, ChIP assay details, and data processing are described in the Materials and methods. Genes are indicated on the top. The grid cells show unadjusted (P), and false discovery rate (FDR) adjusted significance values (Q) for all contrasts shown on the left. None of the contrasts reach the significance threshold at 5% FDR (Q<0.05). (TIF) [file pone.0280645.s010.tif]

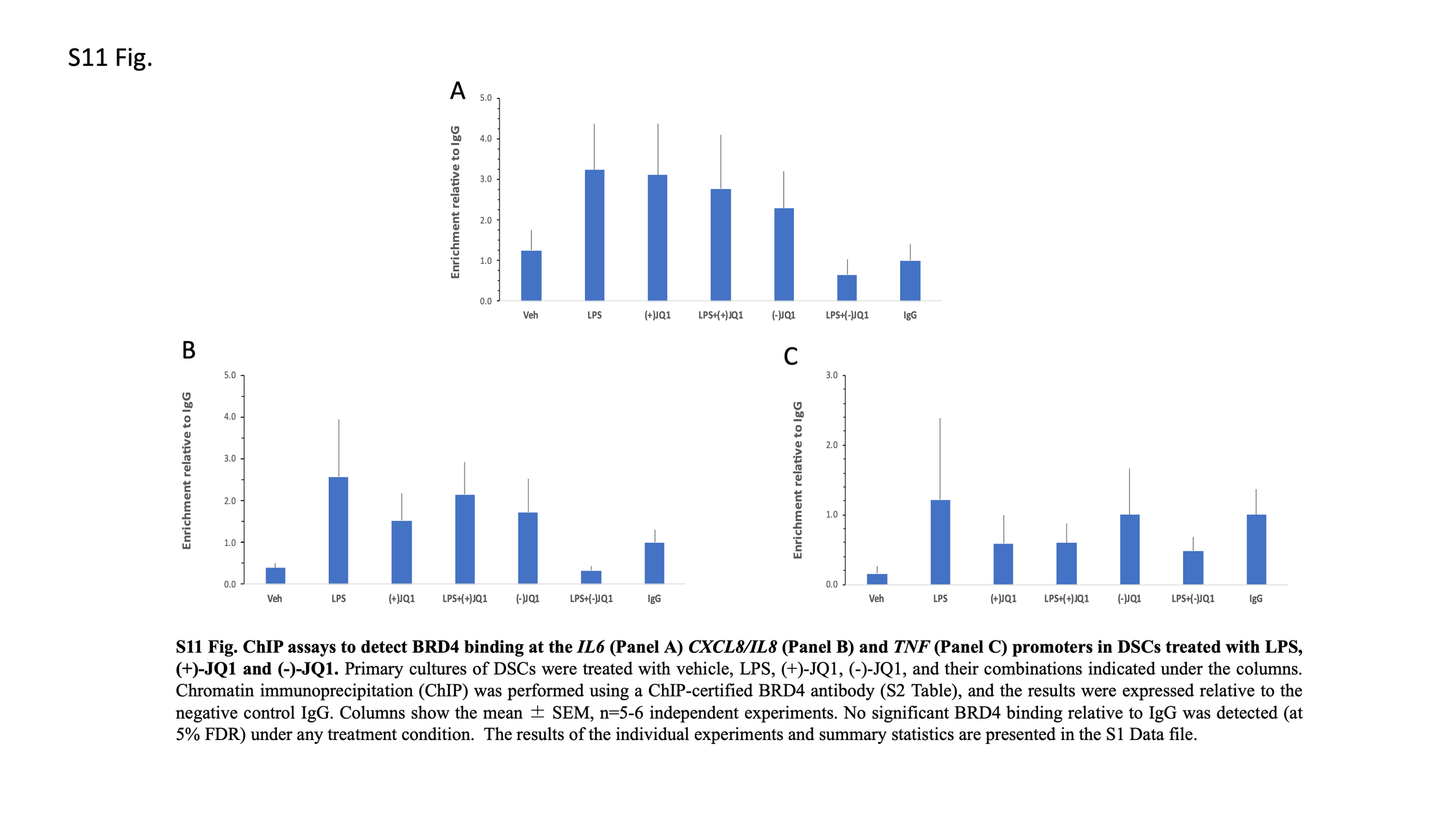

Supplement: S11 Fig — ChIP assays to detect BRD4 binding at the IL6 (Panel A) CXCL8/IL8 (Panel B) and TNF (Panel C) promoters in DSCs treated with LPS, (+)-JQ1 and (-)-JQ1. Primary cultures of DSCs were treated with vehicle, LPS, (+)-JQ1, (-)-JQ1, and their combinations indicated under the columns. Chromatin immunoprecipitation (ChIP) was performed using a ChIP-certified BRD4 antibody (S2 Table), and the results were expressed relative to the negative control IgG. Columns show the mean ± SEM, n = 5–6 independent experiments. No significant BRD4 binding relative to IgG was detected (at 5% FDR) under any treatment condition. The results of the individual experiments and summary statistics are presented in the S1 Data. (TIF) [file pone.0280645.s011.tif]

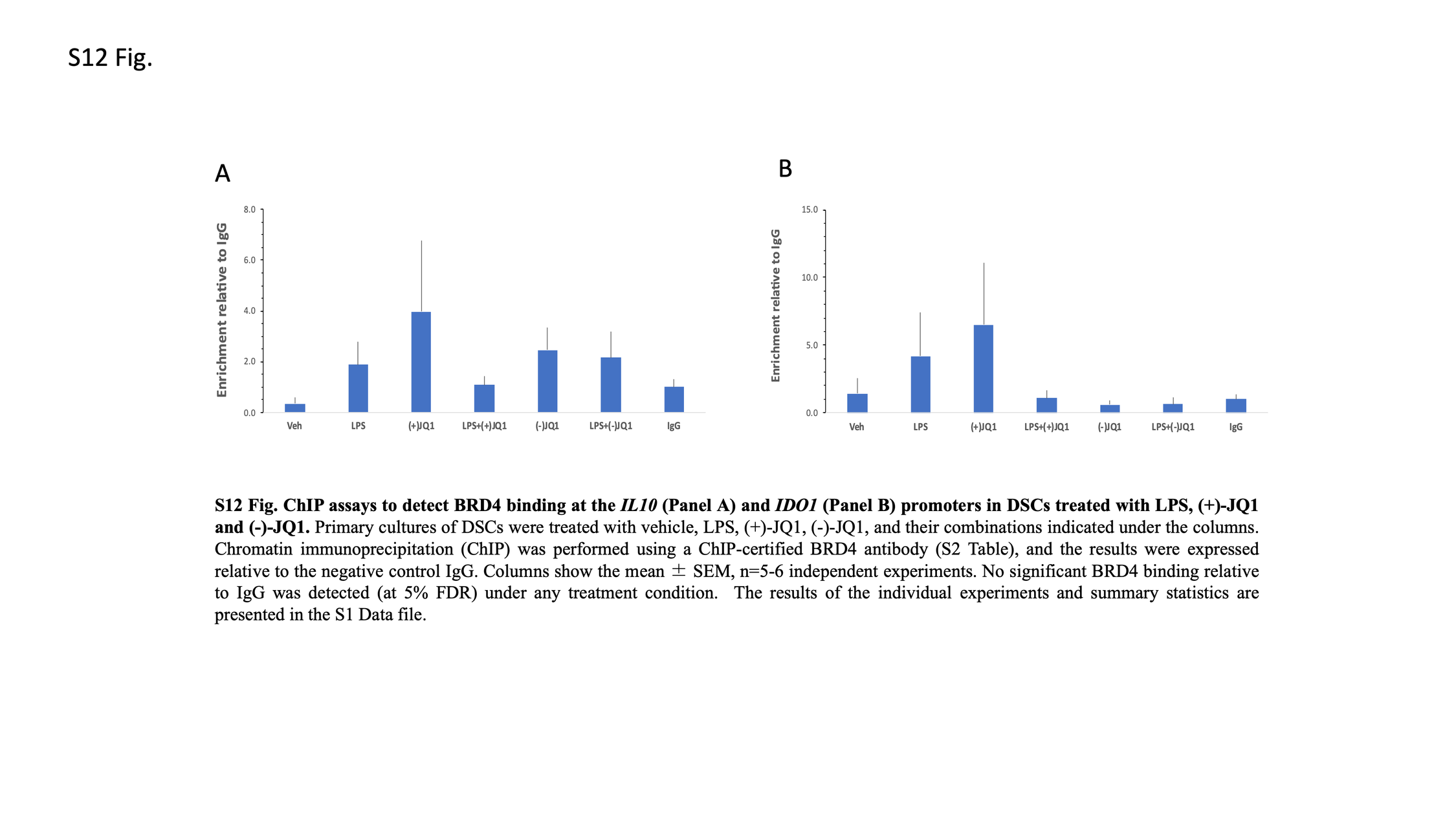

Supplement: S12 Fig — ChIP assays to detect BRD4 binding at the IL10 (Panel A) and IDO1 (Panel B) promoters in DSCs treated with LPS, (+)-JQ1 and (-)-JQ1. Primary cultures of DSCs were treated with vehicle, LPS, (+)-JQ1, (-)-JQ1, and their combinations indicated under the columns. Chromatin immunoprecipitation (ChIP) was performed using a ChIP-certified BRD4 antibody (S2 Table), and the results were expressed relative to the negative control IgG. Columns show the mean ± SEM, n = 5–6 independent experiments. No significant BRD4 binding relative to IgG was detected (at 5% FDR) under any treatment condition. The results of the individual experiments and summary statistics are presented in the S1 Data. (TIF) [file pone.0280645.s012.tif]
