# Supplementary material for: Regulation of inflammatory genes in decidual cells: Involvement of the bromodomain and extra-terminal family proteins
Source: PLoS One. 2023 Mar 10;18(3):e0280645. doi: 10.1371/journal.pone.0280645 (PMC10004631; doi:10.1371/journal.pone.0280645)
Supplement: S1 Table — (DOCX) [file pone.0280645.s013.docx]

| S1 Table. Primer Sequences | | |
| --- | --- | --- |
|  | NCBI GenBank  Accession | Primer Sequence |
| *PTGS1 mRNA* | NM_000962 | Fw: CCA GGA GCT CGT AGG AGA GAA G |
|  |  | Rev: GCA TCA ATG TCT CCA TAC AAT TCC |
| *PTGES mRNA* | NM_007848 | Fw: TGT ACG TGG TGG CCA TCA TC |
|  |  | Rev: GGT TGG CAA AGG CCT TCT TC |
| *PTGS2 mRNA* | NM_000963 | Fw: GAA TCA TTC ACC AGG CAA ATT G |
|  |  | Rev: TCT GTA CTG CGG GTG GAA CA |
| *IL6 mRNA* | NM_000600 | Fw: AAA AAG GCA AAG AAT CTA GAT GCA A |
|  |  | Rev: CAG CAG GCT GGC ATT TGT G |
| *CXCL8/IL8 mRNA* | NM_000584 | Fw: CTT TCT GAT GGA AGA GAG CTC TGT CT |
|  |  | Rev: TCT CAG CCC TCT TCA AAA ACT TCT |
| *TNF mRNA* | NM_000594 | Fw: CCC AGG CAG TCA GAT CAT CTT C |
|  |  | Rev: GGT TTG CTA CAA CAT GGG CTA CA |
| *IL10 mRNA* | NM_000572 | Fw: GTC ATC GAT TTC TTC CCT GTG AA |
|  |  | Rev: TTG GAG CTT ATT AAA GGC ATT CTT C |
| *IDO1 mRNA* | NM_002164 | TGC TTC TGC AAT CAA AGT AAT TCC |
|  |  | CAA AGT GTC CCG TTC TTG CAT |
| *BRD2 mRNA* | NM_005104 | Fw: GGG GCC GAT GAA GAT GAC AA |
|  |  | Rev: CTT TTT GGG GAG CTT GGT GC |
| *BRD3 mRNA* | NM_007371 | Fw: GCT CCA GGA CGT GTT TGA GAT |
|  |  | Rev: GAG CTC TCC TCA CTG CTA CG |
| *BRD4L mRNA* | NM_058243 | Fw: CCT ACT CAA CCG GTC ACC TC |
|  |  | Rev: GTT TTG CTG GGG TGG AGA CT |
| *BRD4S mRNA* | NM_014299 | Fw: CCG AAA CAG GTC CTG CCT AA |
|  |  | Rev: GAG TCC TGT CCC TTT CAC GG |
| *IL6 promoter* | NG_011640 | Fw: CAC CCT CAC CCT CCA ACA AA |
|  |  | Rev: TTC TCT TTC GTT CCC GGT GG |
| *IL8/CXCL8 promoter* | NG_029889 | Fw: CCA TCA GTT GCA AAT CGT GGA AT |
|  |  | Rev: GAG TGC TCC GGT GGC TTT TTA |
| *TNFα/TNF promoter* | NG_007462 | Fw: CGC GAT GGA GAA GAA ACC GA |
|  |  | Rev: TAT ATG TCC CTG GGG CGA GA |
| *IL10 promoter* | NG_012088 | Fw: AGA GAA GGA GGA GCT CTA AGC A |
|  |  | Rev: TCA CCT CTC TGT CCC CCT TT |
| *IDO1 promoter* | NG_028155 | Fw: CCG GCC ACC TGT TTT CAT AG |
|  |  | Rev: ACA AAA GCA TCT CTG TGC ATC AT |
