# Supplementary material for: Regulation of inflammatory genes in decidual cells: Involvement of the bromodomain and extra-terminal family proteins
Source: PLoS One. 2023 Mar 10;18(3):e0280645. doi: 10.1371/journal.pone.0280645 (PMC10004631; doi:10.1371/journal.pone.0280645)
Supplement: S2 Table — (DOCX) [file pone.0280645.s014.docx]

| S2 Table. Antibodies | | |  |
| --- | --- | --- | --- |
| Antibody | Supplier | Cat. No. / Lot No. | Identifier |
| Acetyl Histone- 3 | Millipore | 06-599 / 3198409 | RRID:AB_2115283 |
| Acetyl Histone- 4 | Millipore | 06-598 / 3473490 | RRID:AB_2295074 |
| BRD2 | Bethyl | A302-583A / 06 | RRID:AB_2034829 |
| BRD4 | Bethyl | A700-004 / 03 | RRID:AB_2631885 |
| IgG (Control) | Santa Cruz Biotechnology | SC2027 / H2615 | RRID:AB_737197 |
