# Supplementary material for: Regulation of inflammatory genes in decidual cells: Involvement of the bromodomain and extra-terminal family proteins
Source: PLoS One. 2023 Mar 10;18(3):e0280645. doi: 10.1371/journal.pone.0280645 (PMC10004631; doi:10.1371/journal.pone.0280645)
Supplement: Attachment — Submitted filename: Responses.docx [file pone.0280645.s016.docx]

Responses to comments by the Academic Editor:

*1. We have used the PLOS ONE Style templates for the formatting of the Manuscript as required. We have included a legend section, as requested by Reviewer 1. We have reformatted Figs. 2, 3, 4, 5, 7, 8, 10, 11 following the request by Dr. Maidelyn R. Peregrin from PLOS ONE.*

*2. We have corrected the grant information in the 'Funding Information' and the 'Financial Disclosure' sections.*

*3. The study's minimal underlying data set is provided as the Supporting Information File 'S1 Data'. We are ready to share all data generated in the study without restriction.*

Responses to Review Comments to the Author

Comments by Reviewer #1: "...the organization is confusing". "The Ms must be restyled reporting data in a more readable form." "A legend section is required."

*Responses: We have reorganized the manuscript and restyled the data presented as follows:*

*We have created heat map grids that condense the effects of LPS and BET inhibitor treatments on the expression of target genes. The heat maps facilitate the summary evaluation of response patterns throughout treatments and genes. Significant stimulations and inhibitions are highlighted by colors. Fig. 1 focuses on LPS effects and BET inhibitor effects on basal expressions and LPS effects in BET inhibitor-treated cells. A heat map including all contrasts is presented in S4 Fig. for a fully detailed, comprehensive view.*

*The diagrams displaying mRNA relative abundance data individually for each target gene were grouped into composite figures to reduce the need to navigate within the document. Fig. 2 includes PTGS1, PTGES, and PTGS2 of the prostaglandin synthesis pathway, Fig. 3 includes IL6, CXCL8/IL8, and TNF encoding proinflammatory factors, and Fig. 4 includes IL10, and IDO1, which are considered anti-inflammatory factors.*

*We have also created heat map grids to summarise the effects of LPS and BET inhibitor treatments on acH3 (Fig. 6) and acH4 (Fig. 9) levels at the gene promoters using a similar approach as in Fig. 1. The complete lists of contrasts are presented in S5 Fig., and S6 Fig. Diagrams displaying ChIP signals individually for each target gene were grouped into Figs. 7 and 8 (acH3) and Figs. 10 and 11 (acH4).*

*Grids and composite figures containing the BRD2 and BRD4 ChIP data are presented in the supplementary dataset since no significant effects were found (S7 - S12 Figs.).*

*A legend section is included in the revised manuscript.*

Comments by Reviewer #2:

1. "...they should show cell homogeneity (exclude other cell contamination)..."

"... a thorough check that the biological material they receive are not already contaminated by LPS or mycoplasma..."

*Response: We have examined the homogeneity of the purified decidual cells by flow cytometry. The results are presented in S1 Fig. The flow cytometry analysis shows that the freshly isolated decidual cell population is not contaminated by leukocytes.*

*The cultured decidual cells have been characterized by immunocytochemistry. The results, presented in S2 Fig., demonstrate that the cells pervasively express vimentin, which is an established marker of decidual stromal cells, and do not express CD45, which is a leukocyte common antigen. These results are included in the revised text (Lines 137-139 and 143-145).*

*We agree with the Reviewer that primary decidual cells may be exposed to microorganisms in situ before isolation. We have minimized this risk by collecting decidua tissue from patients without symptoms of intrauterine infection or inflammation and without signs of histological chorioamnionitis. Furthermore, the response of the cultured cells to LPS was robust, arguing against residual LPS stimulation in situ. Nevertheless, potential in situ infectious exposure represents a limitation of the study, which we acknowledge in the revised manuscript (Lines 104-105 and 380-384).*

2. "...an interesting experiment would be to check whether supernatants from LPS-stimulated cells (and controls) could mimic the same effect...

*Response: We agree with the Reviewer that LPS-induced factors may be secreted in the media, potentially mediating or mimicking the effects we report. Identifying the contribution of such factors would be very important. The problem we face here is that the conditioned media still contain LPS requiring additional procedures to differentiate between bona fide LPS actions and LPS-induced mediator effects. One future research direction is to establish assay systems where bona fide LPS and LPS-induced mediator actions are discerned and characterized separately in the conditioned media.*

3. "...a summary scheme would be beneficial"

*Response: The heat map grids described in the responses to Reviewer #1 were designed to serve as summary schemes.*
